# Decreased Blood Levels of Oxytocin in Ketamine-Dependent Patients During Early Abstinence

**DOI:** 10.3389/fpsyt.2018.00633

**Published:** 2018-11-26

**Authors:** Ming-Chyi Huang, Lian-Yu Chen, Hu-Ming Chang, Xiao-Yu Liang, Chih-Ken Chen, Wan-Ju Cheng, Ke Xu

**Affiliations:** ^1^Department of Psychiatry, Taipei City Psychiatric Center, Taipei City Hospital, Taipei, Taiwan; ^2^Department of Psychiatry, School of Medicine, College of Medicine, Taipei Medical University, Taipei, Taiwan; ^3^Psychiatric Research Center, Taipei Medical University Hospital, Taipei, Taiwan; ^4^Institute of Epidemiology and Preventive Medicine, National Taiwan University, Taipei, Taiwan; ^5^Department of Psychiatry, Yale University School of Medicine, New Heaven, CT, United States; ^6^Department of Psychiatry, Chang Gung Memorial Hospital, Keelung, Taiwan; ^7^Chang Gung University School of Medicine, Taoyuan, Taiwan; ^8^Department of Psychiatry, China Medical University Hospital, Taichung, Taiwan; ^9^Department of Public Health, China Medical University, Taichung, Taiwan

**Keywords:** oxytocin, ketamine, dependence, withdrawal, NMDA antagonist

## Abstract

**Background:** Ketamine, an N-methyl-D-aspartate (NMDA) receptor antagonist, is a common drug of abuse worldwide. Existing evidence suggest a disruption of oxytocin system involves in the development of addiction. In this study, we aimed to investigate the role of oxytocin in ketamine addiction by measuring the blood oxytocin levels in ketamine-dependent (KD) patients.

**Methods:** Sixty-five KD patients and 65 controls were enrolled. Fasting plasma levels of oxytocin were determined at baseline and 1 and 2 weeks after ketamine withdrawal. Ketamine use variables, Beck Depression Inventory, Beck Anxiety Inventory (BAI), Visual Analog Scale for craving, and Childhood Trauma Questionnaire-short form were assessed in KD patients.

**Results:** KD patients had significantly lower levels of oxytocin at baseline compared to controls (5.89 ± 2.13 vs. 9.53 ± 4.17 ng/mL, *P* < 0.001). Oxytocin levels increased after one (6.74 ± 2.63, *P* < 0.002) and 2 weeks (6.89 ± 2.69, *P* = 0.01) of withdrawal in KD patient despite the levels were still lower than controls (*P* = 0.001 and 0.002, respectively). The clinical variables did not correlate with baseline oxytocin levels except BAI scores, which showed a negative correlation with the levels (*r* = −0.263; *P* = 0.039).

**Conclusion:** We found a distinctively reduced oxytocin level in KD patients and the level did not normalize after early abstinence. Lower oxytocin might be associated with anxious phenotype of ketamine dependence. These results suggest that oxytocin system dysregulated following chronic ketamine abuse and might provide insight in evaluating the potential therapeutic use of oxytocin for treating ketamine dependence.

## Introduction

Ketamine hydrochloride, a non-competitive antagonist of N-methyl-D-aspartate (NMDA) receptor, has long been used as a short-acting anesthetic agent in humans and veterinary medicine. However, recreational ketamine use has been increased significantly worldwide (1), particularly in East and South-East Asia (2), and constitutes a major challenge to public health (3) because of the association with multi-organ harms (4). Ketamine has become one of the most abused drugs in Taiwan (5). As treatment for ketamine dependence has not yet been available (6), knowledge of the underlying mechanisms that modulates the reward system may provide insight in managing individuals suffering from ketamine dependence.

Emerging evidence suggests that oxytocin is involved in the neuroadaptive processes associated with the development of addiction (7). Oxytocin is a polypeptide hormone synthesized in the magnocellular neurons located within the supraoptic (SON), paraventricular (PVN), and accessory nuclei of the hypothalamus and released by the posterior pituitary gland, acting predominantly upon the oxytocin receptor, which is widely distributed throughout brain circuits related to reward, learning, memory, stress, and addiction (8). Animal studies have demonstrated that oxytocin interferes the development of tolerance to morphine (9) and alcohol (10). It may also have the effects of reducing self-administrative behavior of heroin (9), alcohol (11), methamphetamine (12), and cocaine (13). In reinstatement models of drug relapse, oxytocin was found to suppress the reinstatement of extinguished drug-seeking behavior (13–16). The administration of carbetocin, an oxytocin receptor agonist, prevents reinstatement of conditioned reinforcing effects of morphine (16) and alcohol (14). Further evidence supporting the role of oxytocin in the involvement of addiction is that a negative association of plasma oxytocin levels with novelty-seeking, one of the indicators of addiction, was observed in heroin users (17).

Oxytocin has also been implicated in the regulation of withdrawal symptoms in both animals and humans (18). For example, oxytocin administration attenuates alcohol withdrawal convulsions (19) and blocks the somatic symptoms of nicotine withdrawal (20). In addition, oxytocin has also been reported to ameliorate the severity of acute or protracted opioid withdrawal (9, 16). In human studies, oxytocin was shown to be superior to placebo in reducing alcohol withdrawal symptoms in individuals undergoing alcohol detoxification (21). These observations collectively suggest that oxytocin dysregulation can occur after repeated exposure of drugs of abuse and play a role in the physical and behavioral effects associated with acute drug withdrawal.

Secretion of oxytocin is modulated by several mechanisms, including the regulation via NMDA receptors which are expressed in magnocellular cells of PVN and SON hypothalamic nuclei (22). Oxytocin neurosecretory cells in the nuclei receive a dense glutamatergic innervation, which plays a major role in the control of oxytocin release (23). It has been reported that intraperitoneal injection of a NMDA receptor antagonist (MK-801) decreases plasma concentrations of oxytocin in rats induced by osmotic stimulation (24). MK-801 also impaired the oxytocin responses to emotional stimuli (25). Supporting the link between NMDA and the oxytocinergic systems, one study in zebrafish demonstrated that oxytocin reversed MK-801-induced behavioral deficits in social interaction (26). Therefore, ketamine, as an NMDA antagonist, when being used chronically may alter the expression of oxytocin. To test this hypothesis, this study aimed to explore the difference of oxytocin plasma levels between treatment-seeking ketamine-dependent (KD) patients with the most recent ketamine use in the preceding 24 h and healthy controls. We also explored whether oxytocin level was normalized in the early stage of abstinence in KD patients. Finally, the correlations between oxytocin levels and ketamine use variables (frequency and quantity, craving scores, severity of dependence), depression and anxiety symptoms, and experiences of childhood trauma, which have been associated previously with oxytocin concentrations (27, 28), were also examined.

## Materials and methods

### Participants

This study was carried out in accordance with the recommendations of Institutional Review Board of Taipei City Psychiatric Center of Taipei City Hospital (IRB No: TCHIRB-1030408), with written informed consent from all subjects. All subjects gave written informed consent in accordance with the Declaration of Helsinki. The protocol was approved by the Institutional Review Board of Taipei City Psychiatric Center of Taipei City Hospital. The study was conducted in Taipei City Psychiatric Center of Taipei City Hospital after obtaining the approval of its Institutional Review Board. Treatment-seeking KD patients who voluntarily admitted to an inpatient ward from January 2014 to December 2015 were consecutively screened for the eligibility of enrollment in this study. The inclusion criteria were as follows: (1) age between 18 and 60 years; (2) fulfilling DSM-IV-TR criteria for ketamine dependence as verified by two board-certified psychiatrists; (3) last ketamine use within 24 h prior to admission, validated by self-report and positive in urine toxicology test for ketamine; (4) an ability to read Chinese and provide informed consent. The exclusion criteria were: (1) other substance use disorder (including abuse and dependence) in the past year except nicotine; (2) history of schizophrenia, bipolar disorder, or major depressive disorder, or having been treated with antipsychotics, mood stabilizers (including lithium, valproic acid, carbamazepine, and quetiapine), or antidepressants; (3) history of systemic medical illnesses such as hypertension, metabolic disorders (e.g., diabetes mellitus), or renal or liver diseases; (4) history of head injury, loss of consciousness, or neurological disorders; (5) inability or refusal to provide urine sample. Healthy controls were enrolled from the physical check-up unit in the hospital with inclusion criteria as (1) age between 18 and 60 years; (2) no other substance use disorder (including abuse and dependence) in the past year, except nicotine; (3) no history of a major psychiatric disorder (including schizophrenia, schizoaffective disorder, bipolar disorder, major depressive disorder, or organic mental disorders) screened using the Mini-International Neuropsychiatric Interview (MINI) (29) by a trained psychologist; (4) no known systemic or neurological diseases such as hypertension, metabolic disorders (e.g., diabetes mellitus), or renal or liver diseases; (5) an ability to read Chinese and provide informed consent. All the participants were given a comprehensive description of the study and then recruited after giving written informed consent.

The ward provides inpatient services in a controlled environment for individuals with substance use disorders and conducts various treatment programs, such as individual psychotherapy, group psychotherapy, family counseling, and vocational training, to help patients manage withdrawal symptoms, achieve initial abstinence, and prevent relapse. Every patient received regular drug testing to ensure their status of abstinence. Oral diazepam 20 mg/day were given on the first day of admission, and then gradually tapered off before day 5 for the treatment of withdrawal symptoms, such as anxiety, shaking, sweating, palpitations, and sleep impairment, as has been suggested previously (30–32).

### Clinical assessment

The patients underwent the following assessments in the next morning of admission after the blood sample collection was performed.

1. Ketamine use variables: The severity of ketamine dependence was measured by the Severity of Dependence Scale (SDS), which has been validated previously for the Chinese version (33). A trained research assistant was responsible for collecting the data of socio-demographic characteristics and ketamine use pattern in the past month using Time-line follow-back (TLFB). Average and maximum daily dose of ketamine as well as ketamine using days in the 30 days prior to the survey were recorded.
2. The severity of craving was measured by the Visual Analog Scale (VAS), on which the participants self-rated their level of craving for ketamine use, following a careful explanation from research assistants, by indicating a position along a continuous line between 0 and 100 mm, 0 being no craving and 100 mm being so severe that the subject was unable to resist ketamine if it is available.
3. The severity of depressive and anxiety symptoms over the past 2 weeks was assessed by the Chinese-version of the 21-item Becker Depression Inventory (BDI) (34) and the Chinese-version of the 21-item Becker Anxiety Inventory (BAI) (35).
4. Five categories of childhood trauma, including emotional, physical, and sexual abuse, and emotional and physical neglect, were assessed using the Childhood Trauma Questionnaire-short form (CTQ-SF) (36), with each subscale measured in five items and rated on a five-point Likert scale. Participants with scores exceeding the cutoff point for moderate exposure on each subscale (emotional abuse: ≥13; physical abuse: ≥10; sexual abuse: ≥8; emotional neglect: ≥15; physical neglect: ≥10) were classified as positive for a history of childhood trauma exposure in that category. The CTQ-SF demonstrates good internal consistency and criterion-related validity. The Chinese-version of CTQ-SF has also been validated and shown favorable factor structure and test-retest reliability (37).

### Laboratory assays

Blood sample was collected once in control participants and 3 times in KD patients on the day following admission and after 1 and 2 weeks of ketamine abstinence. The instructions about the process were given on admission and repeated on the evening prior to the day of blood withdrawal. Venous blood samples were collected and placed in ice-cold vacutainer tubes containing EDTA (1 mg/ml of blood) anti-coagulant at 8:00–9:00 a.m. after an overnight fasting. The plasma was separated by centrifugation at 3,000 × g for 15 min at 4°C, and aliquots of plasma were immediately frozen and stored at −80°C until assayed. The range of storage time for plasma samples was from 1 to 1.5 years.

Plasma oxytocin levels were determined using enzyme immunosorbent assay (Catalog number: EKE-051-01, Phoenix Pharmaceuticals, Inc., Burlingame, California, USA). Each plasma sample was assayed in duplicates and the mean of the two measurements was used for analysis. The intra-essay and inter-assay coefficients of variation was 9 and 12%, respectively. The detection range of oxytocin assay were 0–100 ng/ml (provided by the manufacturer). There was no significant cross-reactivity or interference between oxytocin and the analogs observed.

### Statistical analyses

In descriptive statistics, we used Chi-square test for categorical variables and independent *t*-test for numerical to compare demographic variables between KD and control groups.

To examine whether oxytocin differ between healthy controls and KD groups by time, we first performed a two-way ANOVA to compare mean differences among groups, adjusting for age and gender. Here, we assumed oxytocin level with no changes in week 1 and week 2 from baseline for controls.

$$y_{i\;j\;k}=\mu +\tau_{i}+\beta_{j}+\epsilon_{i\;j\;k}\{\begin{array}{c} i=1,2\;\\ j=1,2,3\;\\ k=1,\ldots ,195 \end{array}$$

μ: overall mean effect

τ_*i*_: the effect of case and control $\{\begin{array}{c} case\\ control \end{array}\;$

β_*j*_: the effect of the *j*^*th*^ level of Time $\{\begin{array}{c} baseline\\ \;week1\;\\ week2\; \end{array}\;$

ϵ_*ijk*_: is a random error component.

Pairwise *t*-test was used to test the alterations of oxytocin levels after 1 or 2 weeks of ketamine discontinuation in the KD group. Bivariate correlation analysis was used to estimate the correlations between the clinical variables, such as ketamine use variables, BDI and BAI scores, and total scores or number of types of childhood trauma assessed by CTQ-SF, and laboratory measures. SAS 9.4 (SAS Institute, Cary, NC, USA) was used for all the analyses. Significance level was set at *P* < 0.05.

## Results

### Sample characteristics

A total of 130 participants, including 65 KD patients and 65 age- and gender-matched controls were enrolled. The demographic and clinical characteristics are shown in Table 1. The proportion of tobacco smokers was significantly higher in KD group than control group (*P* < 0.001). All of KD patients had administered ketamine for an extended period of time (7.9 ± 4.3 years), with relatively high doses (average and maximum daily dose: 3.5 ± 2.7 and 7.2 ± 6.1 g, respectively) and high frequency of use (24.3 ± 10.9 days) in the past 1 month. All of them reported the main route of ingestion as snorting without injection. The demographic and clinical assessment data were comparable between those receiving blood withdrawal at week 1 (*N* = 38) and those who did not (*N* = 27).

**Table 1:** Sociodemographic and clinical characteristics in treatment-seeking ketamine-dependent (KD) patients and controls.

	**Controls (*N* = 65)**	**KD patients (*N* = 65)**	***P*-value**
Age, years, mean ± SD	31.3 ± 9.6	30.9 ± 5.7	0.77
Sex, *N* (%)			0.70
Male	44 (67.7%)	46 (70.8%)
Female	21 (32.3%)	19 (29.2%)
Current smoker, N (%)	5 (7.7%)	57 (95.0%)	< 0.001
Ketamine use variables, mean ± SD		
Total years of ketamine use		7.9 ± 4.3
Average daily dose in past 30 days (g/day)		3.5 ± 2.7
Maximum daily dose in past 30 days (g/day)		7.2 ± 6.1
Using days in past 30 days (days)		24.3 ± 10.9
VAS for craving (0-100), mean ± SD		42.4 ± 35.2
SDS scores, mean ± SD		10.2 ± 3.6
BDI scores, mean ± SD		27.0 ± 12.8
BAI scores, mean ± SD		16.7 ± 13.0
CTQ-SF			
Number of trauma types		1.9 ± 1.5
Total scores		61.2 ± 15.0

*BDI, Beck Depression Inventory; BAI, Beck Anxiety Inventory; CTQ-SF, Childhood Trauma Questionnaire-short form; SDS, Severity of Dependence Scale; VAS, Visual Analog Scale for craving*.

### The oxytocin levels in KD patients

**Figure 1:**
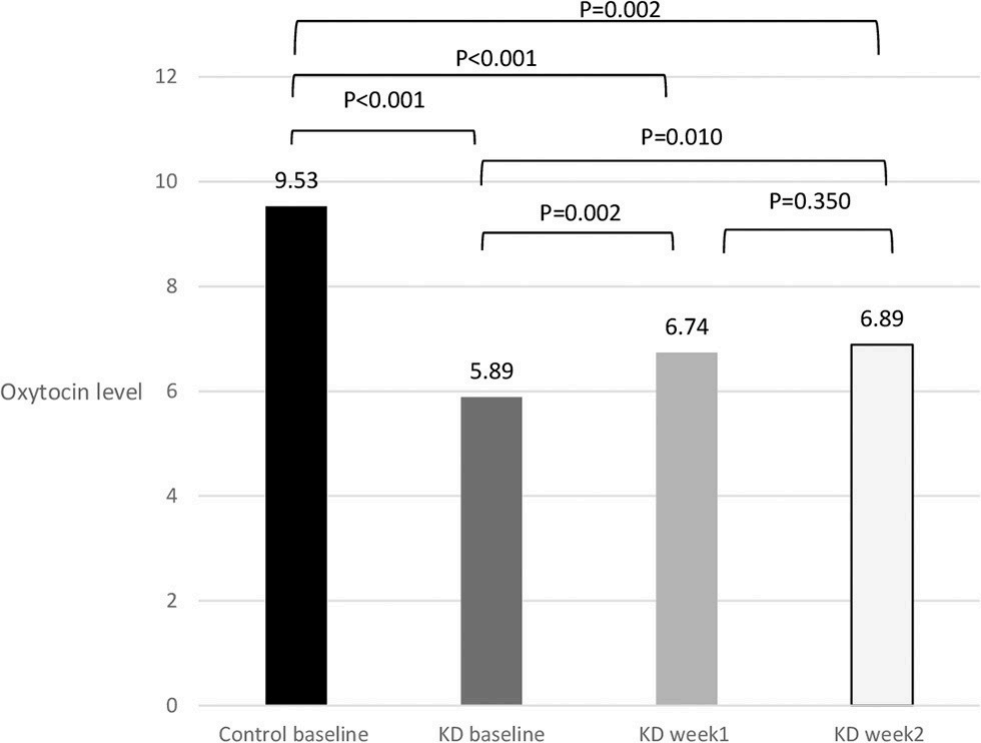
Decreased plasma levels of oxytocin (ng/mL) following 1 and 2 weeks of ketamine discontinuation in ketamine-dependent (KD) patients.

Two-way ANOVA showed that oxytocin level was significantly lower in KD compared to control group. We found no significant difference of oxytocin level by time within KD groups (shown in Figure 1).

In pairwise *t*-tests, we found that KD patients had significantly lower levels of oxytocin at baseline compared to controls (5.89 ± 2.13 vs. 9.53 ± 4.17 ng/mL, *P* < 0.001). Thirty-eight out of 65 patients with KD provided blood samples after 1 week of ketamine abstinence whereas among them 32 patients provided blood after 2 weeks' abstinence. We found oxytocin levels increased after one (6.74 ± 2.63, *P* < 0.002) and 2 weeks (6.89 ± 2.69, *P* = 0.01) following ketamine discontinuation comparing with baseline oxytocin level; however, the oxytocin in week 1 and week 2 among KD patients were still significantly lower than controls (*P* = 0.001 and 0.002, respectively).

### The correlation of oxytocin levels with ketamine use variables, depression or anxiety symptoms, and childhood trauma exposure

The baseline oxytocin levels were not significantly correlated with ketamine use variables, including total years of ketamine use, daily dose and using days in the past 1 month, or VAS of ketamine craving, depression severity, and total scores or number of types of childhood trauma in CTQ-SF (*P*-values: all >0.05) (shown in Table 2). There was a marginal significant correlation between oxytocin levels and SDS scores (*r* = −0.253; *P* = 0.058), suggesting a trend of lower oxytocin levels in patients with higher dependence severity. Noteworthily, anxiety symptoms were negatively correlated with oxytocin levels at baseline (*r* = −0.263; *P* = 0.039).

**Table 2:** The correlation of oxytocin levels at baseline with clinical variables in KD patients.

	***r***	***P*-value**
Ketamine use variables	
Total years of ketamine use	−0.209	0.110
Average daily dose in past 30 days (g/day)	0.081	0.532
Maximum daily dose in past 30 days (g/day)	0.023	0.861
Using days in past 30 days (days)	0.064	0.621
VAS	−0.055	0.673
SDS	−0.253	0.058
BDI	−0.159	0.217
**BAI**	–**0.263**	**0.039**
CTQ-SF	0.013	0.924
Total scores	0.013	0.924
Number of trauma types	−0.025	0.848

*BDI, Beck Depression Inventory; BAI, Beck Anxiety Inventory; CTQ-SF, Childhood Trauma Questionnaire-short form; SDS, Severity of Dependence Scale; VAS, Visual Analog Scale for craving. Bold highlights statistically significant values (p < 0.05)*.

## Discussion

To our knowledge, no studies to date have explored the possible association of oxytocin with chronic NMDA antagonism. Specifically, we found a significantly decreased blood level of oxytocin in patients with ketamine dependence compared to healthy controls. More interestingly, the reduction of oxytocin did not normalize after 2 weeks of withdrawal. Of note, patients with greater anxiety severity were associated with a lower oxytocin level in blood. Our preliminary findings highlight a possibility that chronic ketamine exposure is associated with a down-regulation of oxytocin and such abnormal oxytocin regulation was persistent in early abstinence.

Existing evidence indicates that oxytocin system interferes with different stages of addictive behavior across the addiction cycle (18) by reducing drug consumption, tolerance, and reinforcing effects, suppressing both acute and protracted withdrawal, and inhibiting relapse (9–16). The possible counteracting effect of oxytocin toward addictive behaviors has also been demonstrated in recent clinical studies that proposed oxytocin might serve as a novel treatment for drug abuse (38). Intranasal oxytocin administration has been shown to be beneficial in treating individuals with alcohol (39), cannabis (40), and cocaine use disorders (41), particularly through the effect of craving reduction. Therefore, it is likely that chronic drug abuse causes a neuroadaptaional down-regulation of oxytocin, and administration of oxytocin may compensate the abnormalities and further modulate the neurobehavioral response (7, 18). In light of this point, our results also support a potential role of oxytocin in the mechanisms underlying the development of ketamine addiction. Previous studies showed that NMDA receptors are involved in the release and secretion of oxytocin (22–24). Whilst NMDA receptor activation may enhance oxytocin release, NMDA antagonist treatment has been shown to suppress the release (42) and decrease the blood level of oxytocin (24). In agreement, our patients using ketamine, an NMDA antagonist, chronically similarly displayed a reduced blood level of oxytocin.

We found oxytocin levels in KD patients increased significantly after 1 week of ketamine discontinuation despite the levels were still deficient even after 2 weeks of withdrawal compared to controls. The alterations of oxytocinergic system have been suggested to contribute to withdrawal symptoms of various substances of abuse. For example, Zanos et al. found oxytocin involves the emotional impairment of morphine abstinence in mice and oxytocin administration attenuated the negative emotional consequences (16). Similarly, oxytocin administration was reported to decrease the alcohol withdrawal-induced convulsion (19), somatic symptoms of nicotine withdrawal (20), and morphine withdrawal-related hypothermia and body weight loss (9). In line with the potential advantage of oxytocin in blocking withdrawal, some clinical evidence also showed that in alcohol-dependent individuals undergoing alcohol detoxification, oxytocin treatment was associated with a superior effect over placebo on alcohol withdrawal symptoms blockade and less symptom-triggered benzodiazepine doses needed for withdrawal management (21). Although we did not measure the severity of withdrawal sequentially, it is possible that the clinical improvement of withdrawal symptoms in KD patients was associated with an elevation of oxytocin during withdrawal. Whether there would be normalization after sustained abstinence should be examined in future studies.

In our study, patients with greater anxiety appeared to have lower oxytocin levels. This finding is in line with the current literature revealing that oxytocin is a profound anxiolytic and anti-stress factor of the brain (43). Behavioral data from the oxytocin gene-deleted mice displayed more anxiety-related behaviors and higher stress hormone (corticosterone) secretion after a stressor challenge compared to their wild-type counterparts (44). It is likely that the reduction in oxytocin may contribute to an increased level of anxiety in patients using ketamine chronically. In agreement with this notion, previous evidence demonstrated that oxytocin treatment reduces the anxiety response in rats during cocaine withdrawal (45). However, one recent study found in heroin users the plasma oxytocin levels were instead positively correlated with anxiety scores during acute withdrawal (46). The reasons for the contradictory findings are not clear. Oxytocin is thought to regulate the homeostasis of stress response mechanisms of hypothalamus-pituitary-adrenal (HPA) axis, which becomes dysfunctional following chronic drug abuse and in turn leads to an increased level of anxiety. The interactions with HPA axis are likely to have a critical role in oxytocin's effect to attenuate heightened anxiety during drug withdrawal (16, 18). Moreover, some evidence showed that different classes of drugs of abuse exhibited dissimilar effects on HPA axis activity at doses that reinforce addictive behaviors (47). For example, while ketamine may inhibit the HPA axis activity (47), chronic alcohol consumption produces a potent activation of the HPA axis in humans (48) and active heroin users showed a normalized HPA axis response (49). Given the interplay between oxytocin system and HPA axis, it is likely the expression pattern of oxytocin might differ between various drugs of abuse.

Findings from this study should be interpreted in light of several limitations. First, we only examined the oxytocin following 2 weeks of ketamine withdrawal and did not follow the depressive or anxiety symptoms after baseline. Given the persistent nature of addiction, future studies are required to examine the alterations oxytocin after a longer term of abstinence as well as how it contributes to the emotional disturbances that are commonly associated with addiction. Second, compared to patients with KD, only a small proportion of our controls were smokers and as result the differences of the biomarker levels between groups might be biased. Despite that preclinical studies suggest nicotine can modulate the oxytocin signaling (20, 50), no human studies to date have reported the association of oxytocin with tobacco smoking. In addition, we did not find a significant correlation between oxytocin levels and smoking amount in patients with KD (*P* = 0.22). Therefore, the effect of tobacco smoking on oxytocin levels might be limited. Third, using plasma oxytocin as a surrogate for central oxytocin function is still controversial because peripheral concentrations may also be contributed by peripheral organs including the gastrointestinal tract, heart, and reproductive tract (51). Despite an affirmative conclusion is still lacking, one recent meta-analysis showed a positive association may exist between central and peripheral oxytocin concentrations (52). Last, apart from the effect of chronic ketamine administration on the oxytocin expression, other individual factors may also contribute to the differences in oxytocin system, such as genetic variation, early life adversities, or stressful social experiences, and thus confound the results (53). We were unable to confirm whether the oxytocin expression in KD patients had been different from normal controls before they ingested ketamine, or a causal relationship between heavy ketamine use and deficient oxytocin expression.

In summary, we found that oxytocin levels were distinctively reduced in KD patients compared to controls and the reduction was persistent throughout the first 2 weeks of baseline was observed. In addition, a lower level of oxytocin was associated with greater anxiety symptoms. These results suggest that oxytocin down-regulation may contribute to the neuroadaptational mechanisms of ketamine addiction and a lower oxytocin might be associated with the anxious phenotype of ketamine addiction. Our data may further provide insight into the neurochemical changes of dysregulated oxytocin signaling after chronic NMDA antagonism. Whether there will be a delayed normalization of oxytocin expression after a longer-term of abstinence should be further testified. Given the growing interests in the utility of oxytocin-based therapeutic approaches to drug and alcohol addiction (7, 18), it might be worth examining the relevance of oxyctocin administration to ketamine dependence treatment in future studies.

## Author contributions

M-CH designed the study and wrote the manuscript. L-YC and H-MC recruited participants and assisted in data interpretation. X-YL and W-JC did the statistical analysis and drafted the results section. C-KC assisted with the design of the analysis and provided statistical consultancy. KX revised the final version. W-JC incorporated edits from co-authors and was responsible for communication.

### Conflict of interest statement

The authors declare that the research was conducted in the absence of any commercial or financial relationships that could be construed as a potential conflict of interest.
